# Prevalence of atrial fibrillation on a 24-hour Holter in adult Indians

**DOI:** 10.1016/j.ihj.2024.06.012

**Published:** 2024-06-14

**Authors:** M Srinivasa Rao, Ajit Mullasari, Jagdish S. Hiremath, G. Sengottuvelu, Aparna Jaiswal, Darshan Jhala, Jitendra Singh Makkar, B.C. Kalmath, Bino Benjamin, Annirudha Dharmadhikari, Mihir Tanna, Aziz Khan, Siddhant Jain, K.A. Sambasivam, A. Purnanand, N S Rama Raju, Goutam Sarkar, Hiren Prajapati, Willem J. verberk

**Affiliations:** a Apollo Hospital Hyderguda, Hyderabad, Telangana, India; b Madras Medical Mission, Chennai, Tamil Nadu, India; c Ruby Hall Clinic, Pune, Maharashtra, India; d Apollo Hospital, Greams Road, Chennai, India; e Fortis Escorts Heart Institute, New Delhi, India; f Lilavati Hospital, Mumbai, Maharashtra, India; g Eternal Heart Care Centre and Research Institute, Jaipur, rajasthan, India; h Bombay Hospital Institute of Medical Science & Jupiter Hospital and Horizon Group of Hospital, Thane, Maharashtra, India; i Jubilee Mission Medical College & Research Centre, Thrissur, Kerala, India; j Shree Saibaba Heart Institute and Research Centre, Nashik, Maharashtra, India; k Olympus Hospital, Rajkot, Gujarat, India; l Crescent Hospital & Heart Centre, Nagpur, Maharashtra, India; m Shalby Hospital, Indore, Madhya Pradesh, India; n GKNM Hospital, Coimbatore, Tamil Nadu, India; o Purna Heart Institute, Vijayawada, Andhra Pradesh, India; p KIMS Hospital, Rajahmundry, Andhra Pradesh, India; q RSV Hospital, Kolkata, West Bengal, India; r Department of Medical Affairs, Eris Lifesciences Ltd., Ahmedabad, Gujarat, India; s CARIM School for Cardiovascular Diseases, Maastricht University, Maastricht, the Netherlands

**Keywords:** Holter, Paroxysmal atrial fibrillation, India

## Abstract

**Objective:**

To evaluate paroxysmal atrial fibrillation (AF) prevalence **in Indian adults who completed 24-Hour Holter monitoring.**

**Methods:**

A total of 23,847 patients (36.9 % women) were analyzed for AF duration using a software algorithm.

**Results:**

AF was diagnosed in 4153 (17.4 %) patients with a median AF duration of 13 min and 55 s.

**Conclusion:**

AF prevalence was high and largely untreated. The short duration of AF episodes indicates a low likelihood of detection during clinical visits, highlighting its potential underestimation in Indian healthcare.

## 1 Introduction

Atrial fibrillation (AF) is the most common sustained cardiac arrhythmia and an important risk factor for stroke.^1^ Affecting over 0.5 % of the general population, its prevalence markedly increases with age.

AF frequently goes undetected because approximately one-third of all patients are asymptomatic,^2^ and many patients experience paroxysmal AF,^3^ which is difficult to identify using regular ECG. These factors, together with limited awareness, contribute to the low priority given to AF screening in India.

Nevertheless, in the past decade, the crude prevalence of strokes in India has been substantial; ranging from 44.3 to 559 per 100,000 individuals in various regions of the country.^4^ This high prevalence can be largely attributed to the presence of AF, which increases the risk of stroke by five-fold.^5^

Holter monitoring, could help in detecting previously undiagnosed AF^6^ but is not widely used in India, because of healthcare access issues and the cost of devices. To address this challenge, an Indian pharmaceutical company launched a program to enhance nationwide access to Holter monitoring for doctors and gathered the data, thus creating a valuable dataset.

This study aimed to analyze this dataset to investigate the frequency and distribution of paroxysmal AF in Indian **adults on a 24-h Holter**.

## 2 Methods

Eris lifesciences Ltd (Ahmedabad, Gujarat, India) established and supported a nationwide Holter monitoring service, **covering primary, secondary and tertiary healthcare facilities throughout India.** Data from January 2015 to January 2023 were analysed.

**Holter monitoring, which records continuous ECG data for 24 h,** was referred when deemed necessary by the treating physicians for reasons such as suspected arrhythmia and symptoms including palpitations, giddiness, and syncope (see Supplementary Table 1). Following ethical approval from the Medilink Ethics Committee at Medilink Hospital Research Centre (Ahmedabad, Gujarat, India), retrospective data collection was used (waiver for consent). Clinicians from each center documented this data in case report forms. Additionally, CHA₂DS₂-VASc Score^7^ was calculated, and Schiller Medio Holter monitors (Schiller Medizintechnik GmbH, Baar, Switzerland) recorded ECG for a minimum duration of 24 h.

AF presence was determined by AF episodes lasting at least 30 s, per criteria set by Calkins et al^8^ The automated algorithm (Medio Darwin algorithm) provided by the device's software was utilized for the identification of AF.^9^

Statistical analyses were performed in RStudio 2023.03.1 for Linux, using Chi-square and *t*-tests/Wilcoxon tests as appropriate, with significance set at *p* < 0.05.

## 3 Results

Among 23,847 patients monitored (average age 55.1 ± 17.7 years, 36.4 % female), 4153 (17.4 %) were diagnosed with AF. These diagnosed patients were characterized by older age (58.9 ± 17.5 vs. 54.3 ± 17.5 years), higher male prevalence compared to females (68.4 % vs. 62.6 %), a lower average 24-h heart rate (72.7 ± 15.0 vs. 74.9 ± 13.0 bpm), and lower overall medication intake (Table 1). Among the patients diagnosed with AF, 2763 (66.5 % of AF patients) had a CHA₂DS₂-VASc Score of 1 or higher, as shown in Table 1. The median duration of AF among diagnosed patients was 13 min and 55 s (813 s), accounting for approximately 0.9 percent of the total monitoring time. The majority of patients (n = 17,980 [75.4 %]) underwent Holter monitoring for suspected arrhythmia, followed by symptoms such as palpitations, giddiness, and syncope.

**Table 1:** patient characteristics of patients with (YES) and without (NO) atrial fibrillation.

	YES (N = 4153)	NO (N = 19,694)	Total (N = 23,847)	p value
Female Gender	1311 (31.6 %)	7365 (37.4 %)	8676 (36.4 %)	<0.001
Maximal heart rate at night	194 (5.4 %)	779 (4.4 %)	973 (4.5 %)	0.009
Age (years)	58.9 (17.9)	54.3 (17.5)	55.1 (17.7)	<0.001
CHA₂DS₂-VASc Score				<0.001
−0	1390 (33.5 %)	7042 (35.8 %)	8432 (35.4 %)	
−1	1424 (34.3 %)	7471 (37.9 %)	8895 (37.3 %)	
−2	964 (23.2 %)	3491 (17.7 %)	4455 (18.7 %)	
- ≥3	375 (9.0 %)	1690 (8.6 %)	2065 (8.7 %)	
Smoking	31 (0.7 %)	130 (0.7 %)	161 (0.7 %)	0.537
Diabetes	177 (4.3 %)	1057 (5.4 %)	1234 (5.2 %)	0.003
Hypertension	357 (8.6 %)	2564 (13.0 %)	2921 (12.2 %)	<0.001
Dyslipidemia	51 (1.2 %)	187 (0.9 %)	238 (1.0 %)	0.101
Chronic Kidney Disease	8 (0.2 %)	16 (0.1 %)	24 (0.1 %)	0.040
Myocardial Infarction	2 (0.0 %)	22 (0.1 %)	24 (0.1 %)	0.240
Stroke	3 (0.1 %)	30 (0.2 %)	33 (0.1 %)	0.207
Peripheral artery disease	0 (0.0 %)	1 (0.0 %)	1 (0.0 %)	0.646
Valvular Heart Disease	3 (0.1 %)	4 (0.0 %)	7 (0.0 %)	0.076
Angioplasty	22 (0.5 %)	84 (0.4 %)	106 (0.4 %)	0.364
Bypass	15 (0.4 %)	51 (0.3 %)	66 (0.3 %)	0.254
Pacemaker	1 (0.0 %)	20 (0.1 %)	21 (0.1 %)	0.126
24-h HR (BPM)	72.7 (15.0)	74.9 (13.0)	74.5 (13.4)	<0.001
Daytime HR (BPM)	75.4 (15.8)	77.7 (13.7)	77.3 (14.1)	<0.001
Nighttime HR (BPM)	68.2 (14.9)	69.8 (12.7)	69.5 (13.1)	<0.001
Anti-hypertensive drugs	542 (13.1 %)	3325 (16.9 %)	3867 (16.2 %)	<0.001
Anti-diabetes drugs	206 (5.0 %)	1250 (6.3 %)	1456 (6.1 %)	<0.001
Lipid lowering agents	356 (8.6 %)	2193 (11.1 %)	2549 (10.7 %)	<0.001
Anti-arrhythmia drugs	0 (0.0 %)	16 (0.1 %)	16 (0.1 %)	0.066
Diuretics	65 (1.6 %)	358 (1.8 %)	423 (1.8 %)	0.262
Beta-blockers	195 (4.7 %)	1446 (7.3 %)	1641 (6.9 %)	<0.001
ACE-inhibitors	74 (1.8 %)	396 (2.0 %)	470 (2.0 %)	0.335
Calcium channel blockers	118 (2.8 %)	792 (4.0 %)	910 (3.8 %)	<0.001
Angiotensin receptor blockers	284 (6.8 %)	1729 (8.8 %)	2013 (8.4 %)	<0.001
Anti-platelet drugs	285 (6.9 %)	1745 (8.9 %)	2030 (8.5 %)	<0.001
BB and/or CCB	285 (6.9 %)	2042 (10.4 %)	2327 (9.8 %)	<0.001
BB and/or CCB and/or APD	466 (11.2 %)	2914 (14.8 %)	3380 (14.2 %)	<0.001

### 3.1 Age-related variations in atrial fibrillation occurrence

**Fig. 1:**
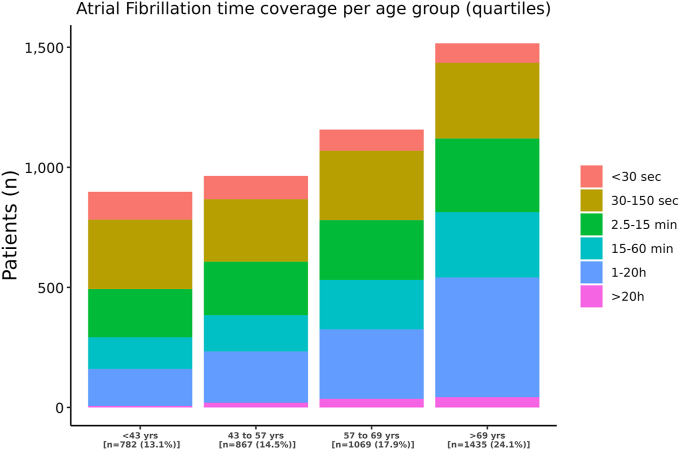
The prevalence of atrial fibrillation (AF) durations across four quartiles of age cohorts in patients diagnosed with AF. The colors within each bar represent the duration of their AF episodes. Below the bars, the numbers on the *x*-axis indicate the average age, along with the number (n) and percentage (%) of AF patients per age group. (For interpretation of the references to colour in this figure legend, the reader is referred to the web version of this article.)

Fig. 1 shows a significant increase in AF prevalence across age groups, from 782 patients (13.1 %) in the youngest quartile (mean age 30.8 ± 8.9 years) to 1435 patients (24.1 %) in the oldest quartile (mean age 76.2 ± 5.7 years). Additionally, the frequency and duration of AF episodes showed exponential increases with advancing age.

## 4 Discussion

The study found a high prevalence of AF, with 17 % of patients diagnosed and a median AF duration of approximately 14 min. Older age, male gender and higher total medication usage were associated with AF. Remarkably, the prevalence of diagnosed hypertension and diabetes was found to be lower in patients with AF, compared to those without AF. Two-thirds of AF patients had a CHA₂DS₂-VASc Score of 1 or higher, indicating an increased risk of stroke or thromboembolism and highlighting potential undertreatment.

### 4.1 Atrial fibrillation and risk factors

Earlier publications identified hypertension as a pivotal risk factor for AF, affecting up to 90 % of individuals in AF research (Manolis et al, 2012). Furthermore, a comprehensive meta-analysis showed that diabetes has a significant association with paroxysmal AF.^10^ Hypertension and diabetes closely relate to AF through pathways like cardiac remodeling and oxidative stress that increase AF risk.^11^

Contrarily, our study observed that the prevalence of hypertension and diabetes was higher in patients without AF than in those with AF. This finding does not necessarily mean these conditions are less common among AF patients. Instead, it may indicate that these patients have not yet been diagnosed, as blood glucose and blood pressure measurements were not included in this study. Therefore, we hypothesize that AF patients relatively more frequently sought care for immediate cardiac-related issues than for diabetes and hypertension, compared to non-AF patients.

### 4.2 Atrial fibrillation and episode duration

Following the Heart Rhythm Society's guideline, which requires at least 30 s of AF on an ECG for diagnosis,^8^ this study identified many patients with short-duration AF episodes. The median AF duration was under 15 min and 38 % (1559 patients) of those diagnosed experienced AF for less than 5 min. This raises questions on the clinical significance of short AF episodes and the sensitivity of current diagnostic criteria.

Short AF episodes are clinically significant because they often indicate more frequent AF occurrences, not detected by 24-h Holter monitoring.^12^ Furthermore, early detection of AF is crucial since AF is progressive, worsening over time and leading to more severe complications through atrial remodeling.^13^ Thus, early detection and treatment are essential in preventing strokes, decreasing AF-related mortality, alleviating symptoms, and decreasing the incidence of heart failure.^14^

Finally, even brief AF episodes significantly increase cardiovascular risk. Borani et al found that an AF duration of at least 5 min was associated with an increased risk of ischemic stroke, evidenced by a HR of 1.76 (95 % CI: 1.02–3.02, *p* = 0.041) during a median follow-up of 24 months.^15^

### 4.3 Strengths and limitations

To our knowledge, this is the largest study in India utilizing 24-h Holter screening, with comprehensive documentation of medication intake and patient characteristics enabling CHA₂DS₂-VASc Score calculations. This offers valuable insights into AF management in the Indian healthcare setting.

The study, however, has limitations: it primarily included patients presenting symptoms or with known cardiovascular risks and was not specifically designed for AF detection. Logistical limitations prevented manual verification of individual patient Holter reports in this study. Therefore, we relied on automated ECG software analysis, potentially leading to false-positive findings. Nonetheless, it is noteworthy that the majority of patients diagnosed with AF exhibited multiple episodes exceeding the 30-s threshold. The accuracy of the Medio Darwin algorithm for detecting AF in Holter recordings has been evaluated, demonstrating a sensitivity of 93.9 % and a specificity of 99.2 %.^9^ Additionally, the baseline characteristics are derived from the data clinicians entered, based on the information available to them at the time of Holter referral. Consequently, the prevalence of certain comorbidities, as shown in Table 1, may have been underestimated.

## 5 Conclusion

This study revealed a high prevalence of paroxysmal AF, with median episodes under 15 min. These findings underscore the limitations of standard ECG and the potential of Holter monitoring to enhance AF detection in the Indian healthcare system.

## Declaration of competing interest

The authors declare the following financial interests/personal relationships which may be considered as potential competing interests:Willem verberk reports financial support was provided by Eris Lifesciences Pvt Ltd. corresponding author is an employee of Microlife corporation If there are other authors, they declare that they have no known competing financial interests or personal relationships that could have appeared to influence the work reported in this paper.
