# Supplementary material for: Prevalence of atrial fibrillation on a 24-hour Holter in adult Indians
Source: Indian Heart J. 2024 Jun 14;76(3):218–20. doi: 10.1016/j.ihj.2024.06.012 (PMC11328999; doi:10.1016/j.ihj.2024.06.012)
Supplement: Multimedia component 1 [file mmc1.docx]

Supplement Table 1, Reasons for 24-h Holter ECG monitoring separated for patients with (AF+ ) and without (AF-) atrial fibrillation

|  | AF+ (N=4153) | AF- (N=19694) | Total (N=23847) | p value |
| --- | --- | --- | --- | --- |
| Suspected Arrhythmia | 3077 (74.1%) | 14903 (75.7%) | 17980 (75.4%) | 0.031 |
| Palpitation | 835 (20.1%) | 3580 (18.2%) | 4415 (18.5%) | 0.004 |
| Giddiness | 123 (3.0%) | 479 (2.4%) | 602 (2.5%) | 0.048 |
| Syncope | 103 (2.5%) | 500 (2.5%) | 603 (2.5%) | 0.827 |
| Stroke | 10 (0.2%) | 77 (0.4%) | 87 (0.4%) | 0.145 |
| Blood pressure related | 2 (0.0%) | 12 (0.1%) | 14 (0.1%) | 0.757 |
| Breathing problems | 0 (0.0%) | 17 (0.1%) | 17 (0.1%) | 0.058 |
| Cardiac abnormalities | 1 (0.0%) | 16 (0.1%) | 17 (0.1%) | 0.210 |
| Chest pain | 2 (0.0%) | 110 (0.6%) | 112 (0.5%) | < 0.001 |
